# Second harmonic generation imaging of corneal stroma after infection by *Pseudomonas aeruginosa*

**DOI:** 10.1038/srep46116

**Published:** 2017-04-11

**Authors:** Danielle M. Robertson, Nathan A. Rogers, W. Matthew Petroll, Meifang Zhu

**Affiliations:** 1 Department of Ophthalmology, The University of Texas Southwestern Medical Center, Dallas, TX, USA.

## Abstract

*Pseudomonas aeruginosa* is a pathogenic gram-negative organism that has the ability to cause blinding corneal infections following trauma and during contact lens wear. In this study, we investigated the directional movement and orientation of an invasive corneal isolate of *P. aeruginosa* in the corneal stroma during infection of *ex vivo* and *in vivo* rabbit corneas using multiphoton fluorescence and second harmonic generation (SHG) imaging. E*x vivo,* rabbit corneas were subject to three partial thickness wounds prior to inoculation. *In vivo*, New Zealand white rabbits were fit with *P. aeruginosa* laden contact lenses in the absence of a penetrating wound. At all time points tested, infiltration of the corneal stroma by *P. aeruginosa* revealed a high degree of alignment between the bacteria and collagen lamellae *ex vivo* (p < 0.001). *In vivo, P. aeruginosa* traveled throughout the stroma in discrete regions or bands. Within each region, the bacteria showed good alignment with collagen lamellae (P = 0.002). Interestingly, in both the *in vitro* and *in vivo* models, *P. aeruginosa* did not appear to cross the corneal limbus. Taken together, our findings suggest that *P. aeruginosa* exploits the precise spacing of collagen lamellae in the central cornea to facilitate spread throughout the stroma.

*Pseudomonas aeruginosa* (PA) is an opportunistic, pathogenic gram-negative organism. In western countries, PA is the leading causative agent in all reported cases of infectious keratitis^1^,^2^,^3^,^4^,^5^,^6^,^7^. By itself, PA is unable to penetrate the healthy cornea. Trauma, pre-existing ocular disease, or contact lens wear are required^1^. The pathogenesis of PA-mediated infectious keratitis is multifactorial and requires a synergistic breach of host defenses^8^,^9^,^10^,^11^,^12^,^13^,^14^. These include bypassing the antibacterial components of the tear fluid and evading the robust host inflammatory response^14^,^15^,^16^,^17^. In addition to the innate immune system, physical barriers exist to block the spread of PA into the cornea^18^,^19^,^20^. At the corneal surface, tight junctions form an impenetrable seal to invading microorganisms^18^,^21^,^22^. Once the epithelial barrier becomes breached however, PA can rapidly invade corneal epithelial cells, where it is protected from clearance by phagocytic inflammatory cells, and is able to traverse the full thickness of the corneal epithelium^23^,^24^,^25^.

Most animal models of PA-corneal infection require a penetrating wound through the basal lamina, the last line of defense against PA and other bacteria from entering the stroma. In the stroma, maintenance of a precise arrangement of collagen lamellae is essential to preserve vision^26^,^27^,^28^. During infection, both bacterial and neutrophil derived proteases in the stroma induce damage that can lead to corneal ulceration, eventual scarring and in some cases, severe permanent loss of corneal clarity. Understanding the pathobiology that leads to disruption of this unique architecture and subsequent opacification of the stroma is essential to mitigate and prevent disease and preserve vision.

Second harmonic generation (SHG) imaging is a form on non-linear optical microscopy that allows for the visualization of fibrillar collagen *in situ*^27^,^29^. In the cornea, SHG imaging has been used to study the normal lamellar architecture in the stroma and more recently, has been used to show how the lamellar architecture is altered during wound healing, following surgery, and in corneal disease^28^,^30^,^31^,^32^,^33^,^34^,^35^,^36^,^37^. Recent work has reported on the use of SHG imaging to investigate changes in the corneal stroma during bacterial infection and showed a corresponding loss of the SHG signal^36^. One potential drawback to this study however, was that the bacteria were not fluorescently labelled, therefore no direct correlations between bacterial infection and alterations in the SHG signals could be drawn. In another study by Tam and colleagues, a unique linear pattern of fluorescently labelled PA within the cornea was observed. While this was suggestive of alignment with collagen lamellae, they were unable to confirm it due to the reflection imaging technique that was used^38^.

In the present study, we sought to systematically evaluate changes in the forward and backward SHG signals using a combination of SHG imaging and multiphoton fluorescence microscopy. To accomplish this, we used an invasive clinical isolate of *Pseudomonas aeruginosa* that we have previously shown can readily infect the rabbit cornea during contact lens wear^39^. Using *ex vivo* and our *in vivo* rabbit cornea infection models, we show, for the first time, that an invasive strain of PA rapidly spreads through the corneal stroma by moving along the collagen lamellae and that SHG signals are not lost until severe infection and liquefactive degradation of collagen occurs. Based on our findings, we further hypothesize that differences in the organization of the lamellae in the sclera and limbus compared to the cornea may contribute to a natural innate barrier that functions to prevent the spread of microorganisms from the conjunctiva into the cornea. This may explain why it is exceedingly rare to see PA infect the limbus.

## Results

**Figure 1:**
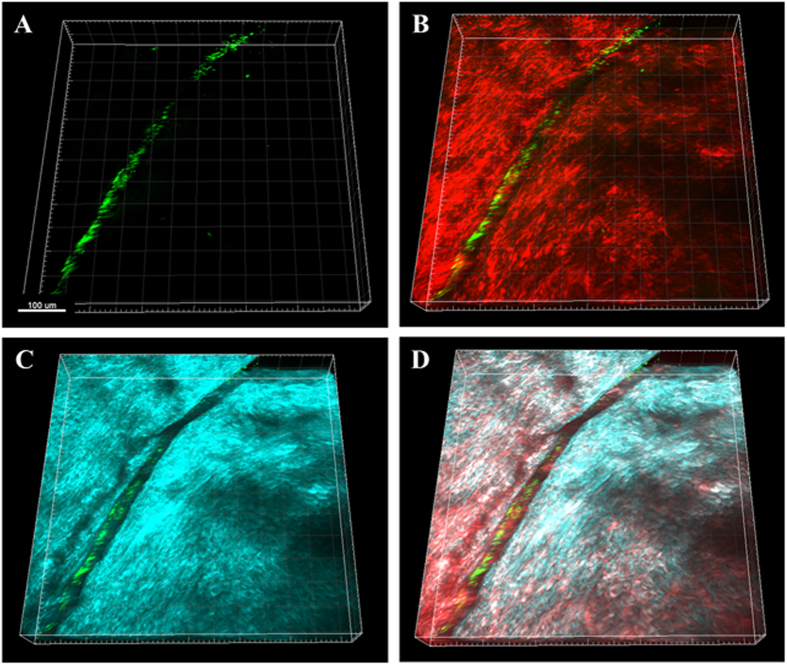
PA localization in the rabbit cornea 2 hours post-inoculation showing PA localization along the wound margin. (**A**) 3-dimensional image showing the distribution of PA (green) in the wounded cornea. (**B**) 3-dimensional image showing the overlay of PA (green) and the forward SHG signal (red). (**C**) 3-dimensional image showing the overlay of PA (green) and the backward SHG signal (teal). (**D**) Combined 3-dimensional image of PA (green), forward SHG signal (red) and backward SHG signal (teal). Images representative of at least 3 repeated experiments. Scale bar: 100 μm.

**Figure 2:**
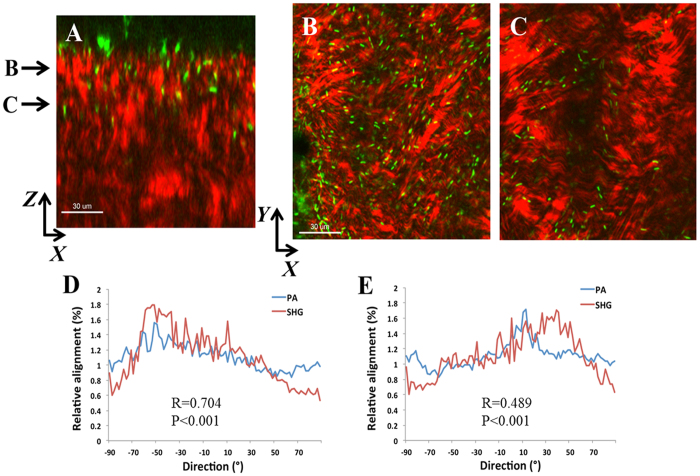
PA localization and orientation in the rabbit cornea 6 hours post-inoculation. (**A**) *XZ* slice showing depth of penetration at 6 hours between collagen fibrils (forward signal in red, PA in green). Scale bar: 30 μm. (**B** and **C**) *XY* slices that correspond to depth arrows in A showing the linear arrangement of PA (green) along the red collagen lamellae. (**D** and **E**) The relationship between the orientation of PA and the collagen fibrils was calculated from the total image (1024 × 1024 pixels). There was a significant correlation in directionality between PA and fibril orientation at all depths, although the correlation was decreased deeper in the stroma due to few bacteria being present (R = 0.704 ± 0.069, P < 0.001 for plane B and R = 0.489 ± 0.059, P < 0.001 for plane C; n = 3 images for each plane). Data representative of at least 3 repeated experiments. Scale bar: 30 μm.

*Ex vivo* rabbit corneas were imaged with SHG multiphoton microscopy. SHG forward and backward signals were detected throughout the normal rabbit corneal stroma (Supplementary Figure 1). Two hours post-inoculation of a wounded cornea showed GFP-labelled PA across the surface of the cornea that appeared to be oriented along the margin of the incisional wound (Fig. 1). At 6 hours post-inoculation, PA began penetrating into the wound margin (Fig. 2A). PA was visible within the superficial corneal stroma, showing a linear arrangement with the forward SHG signal indicating alignment with the collagen lamellae (Fig. 2B and C). Directionality assessment of the orientation of PA at 6 hours showed a strong relationship between the orientation of the PA and the collagen lamellae (R = 0.704 ± 0.069, P < 0.001) in superficial layers (Fig. 2D). This relationship was maintained as PA began to penetrate deeper into the stroma (R = 0.489 ± 0.059, P < 0.001), although the correlation was not as strong due to fewer bacteria being present in the deeper layer at this early time point (Fig. 2E). There was a significant difference in the correlation coefficient between the anterior and deeper planes (P = 0.015, n = 3 images per plane, Supplementary Figure 2A).

**Figure 3:**
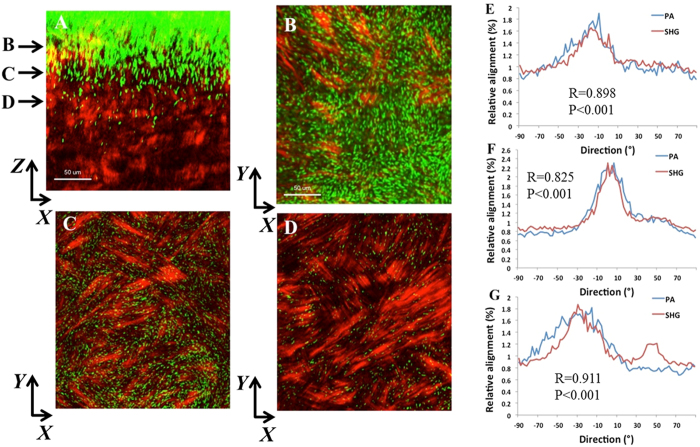
PA localization and orientation in the rabbit cornea 18 hours post-inoculation. (**A**) *XZ* slice showing density of PA and depth of penetration at 24 hours (forward SHG signal in red, PA in green). Scale bar: 50 μm. (**B**–**D**) *XY* slices that correspond to depth arrows in A showing the linear arrangement between PA and collagen lamellae. Scale bar: 50 μm. (**E**–**G**) Relative alignment between PA and collagen fibrils. Results calculated from the total image (1024 × 1024 pixels). There was a significant correlation in directionality at all depths. Three images from each depth plane were analyzed. Results calculated from the total image (1024 × 1024 pixels). (**E**) R = 0.898 ± 0.075, P < 0.001. (**F**) R = 0.825 ± 0.017, P < 0.001). (**G**) R = 0.911 ± 0.006, P < 0.001). Data representative of at least 3 repeated experiments. Scale bar 30 μm.

**Figure 4:**
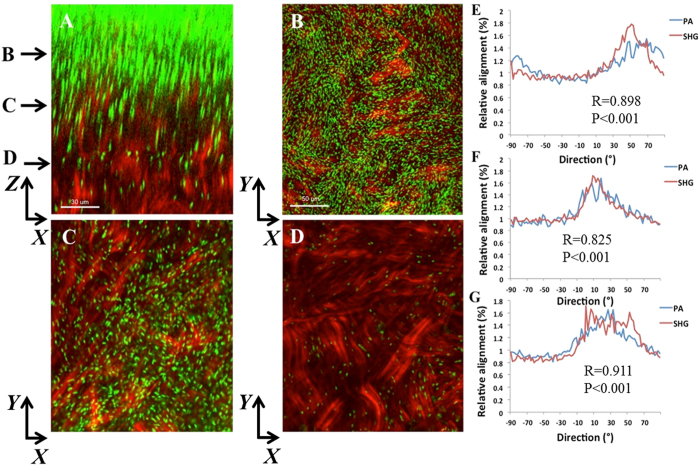
PA localization and orientation in the rabbit cornea 24 hours post-inoculation. (**A**) *XZ* slice showing density and depth of penetration at 24 hours (forward SHG signal in red, PA in green). Scale bar: 30 μm. (**B**–**D**) *XY* slices that correspond to depth arrows in A. Scale bar: 50 μm. (**E**–**G**) The relative alignment between PA and collagen fibrils. (**E**) R = 0.782 ± 0.056, P < 0.001; (**F**) R = 0.739 ± 0.025, P < 0.001; (**G**) R = 0.870 ± 0.072, P < 0.001). Results calculated from the total image (1024 × 1024 pixels). Three images from each depth plane were analyzed. Data representative of at least 3 repeated experiments.

At later time points, PA continued to move vertically into the stroma. At 18 hours post-infection, dense infiltration was evident at the surface (Fig. 3A–D). A strong correlation between the orientation of PA and the forward SHG signal was detected at the anterior (R = 0.898 ± 0.075, P < 0.001, Fig. 3E), mid-anterior (R = 0.825 ± 0.017, P < 0.001, Fig. 3F), and mid-stromal level (R = 0.911 ± .0.006, P < 0.001, Fig. 3G). There was no significant difference between correlation coefficients at any of the planes (Supplementary Figure 2B). Similarly, 24 hours post-infection, traversal through the corneal stroma had deepened and showed a strong relationship between the orientation of PA and the forward SHG signal in the anterior and mid-stromal regions (Fig. 4, R = 0.782 ± 0.056 and R = 0.739 ± 0.025, P < 0.001 for both, respectively). In the deeper cornea, the relationship was somewhat weaker due to fewer PA being present in that region (R = 0.870 ± 0.072, R = 0.001). Again, there was no difference in the correlation coefficient between planes (Supplementary Figure 2C). A comparison on all planes at each incubation time showed a significant difference, with the lowest correlation at 6 hours, when fewest bacteria were present compared to 18 or 24 hours (P < 0.001). There was no difference between 18 and 24 hours (Supplementary Figure 2D; Supplementary movies shown in 4A and AB).

**Figure 5:**
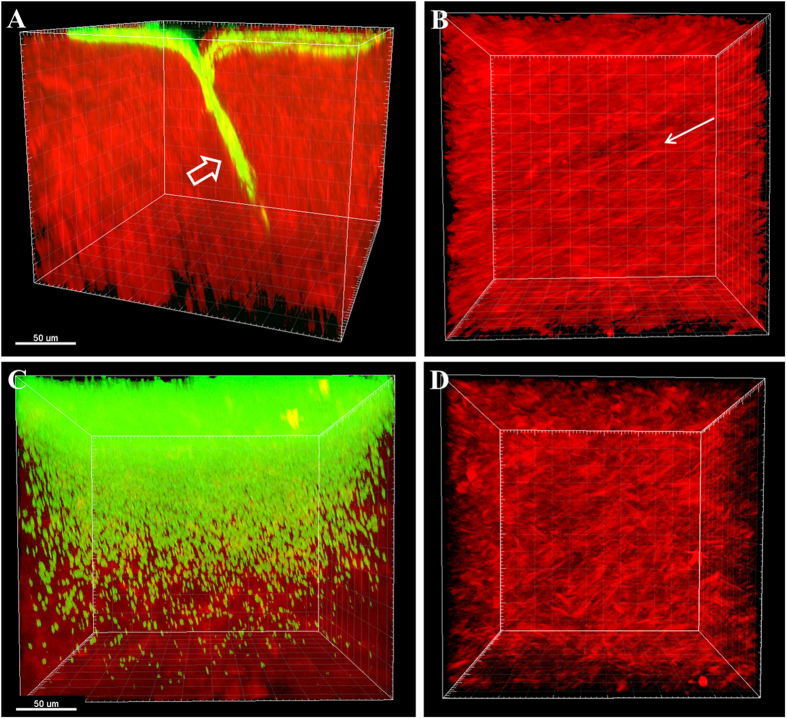
PA spread through the limbus and central cornea at 48 hours. (**A**) 3-dimensional image showing PA invasion into the limbus at the incision site at 48 hours. PA (green) and forward SHG signal (red). PA localized along the apical side of cornea and moved directly in to the wound margin (open arrow). (**B**) *En face* image of apical side of cornea showing distribution of PA across the surface of the collagen. Note the linear arrangement of collagen lamellae (arrow). (**C**) Spread of PA through the corresponding central cornea of the same eye at 48 hours. Massive distribution of PA was seen throughout the stroma. (**D**) *En face* image of the apical surface of the stroma without the green channel showing the characteristic crosshatch appearance of the central cornea, as opposed to the linear arrangement of collagen in the limbus (**B**). Data representative of at least 3 repeated experiments. Scale bar: 50 μm.

To evaluate differences between the central cornea and limbus, an incision was made in central cornea that extended into the limbal region. 48 hours after inoculation, PA was visible across the surface of the limbus and deep within the wound margin, but had not spread throughout the cornea (Fig. 5A). *En face* imaging of the forward SHG signal showed the characteristic linear arrangement of collagen at the limbus (Fig. 5B). In contrast to the limbus, analysis of the central cornea revealed robust spread of throughout the stroma (Fig. 5C). Imaging of the forward SHG signal showed the typical cross-hatch lamellar structure of the central cornea (Fig. 5D).

**Figure 6:**
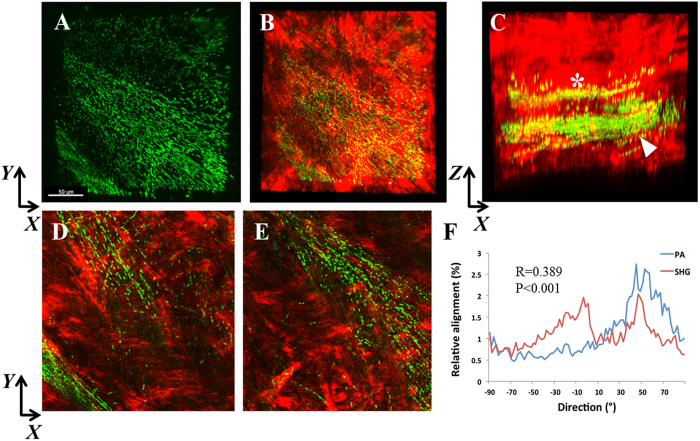
SHG imaging of the infected rabbit cornea *in vivo*. (**A**) *XY* slice showing PA (green) localization within bands of collagen in an apparent linear structure. (**B**) PA combined with forward SHG signal (red). (**C**) *XZ* slice showing the distribution of PA within discrete bands throughout the mid-stromal region. A smaller band (*) was apparent in the mid-stroma, and a larger band (arrowhead) was present more posteriorly. (**D**) *XY* slice corresponding to asterisk region in C showing linearized organization of PA. (**E**) *XY* slice corresponding to arrowhead region in C showing the linearized organization of PA. Scale bar: 50 μm. (**F**) Directionality analysis of segmented region (256 × 256 pixels) isolating the areas of banding showed a small, but significant correlation between the relative alignment of PA and collagen fibrils (R = 0.389 ± 0.171, P < 0.001). Depending on the plane depth, R values ranged from 0.209 to 0.668. In areas outside of the yellow banding, no significant correlation was detected. Data representative of 3 independent rabbits.

**Figure 7:**
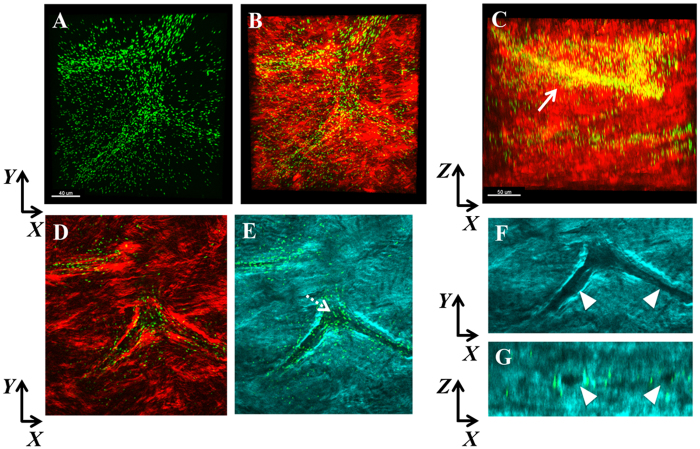
SHG imaging of the infected rabbit cornea *in vivo* showing co-localization of PA with what appears to be a stromal nerve. (**A**) *XY* slice showing the distribution of PA (green). (**B**) *XY* slice showing merged imaged of PA with forward SHG signal (red). Scale bar: 40 μm. (**C**) Representative *XZ* slice of merged imaged showing the spatial distribution of PA within the stroma. Scale bar: 50 μm. (**D**) *XY* slice showing PA combined with the forward SHG signal at a different depth than in A. (**E**) *XY* slice showing PA combined with the backward SHG signal at the corresponding depth as in D. Dotted arrow shows the complete absence of SHG signal. (**F**) Zoomed image of E without PA showing discrete regions without any backward SHG signal (arrowheads). (**G**) *XZ* slice showing discreet circular holes devoid of backward SHG signal that correspond to the regions in (**F**).

To test the relationship between PA orientation and collagen lamellae *in vivo,* rabbits were fit with high oxygen permeable, contemporary rigid gas permeable contact lenses inoculated with PA. No penetrating wound through the basement membrane was required for this model. Biomicroscopic images showing an uninfected rabbit cornea and two representative PA-infected rabbit corneas *in vivo* at 24 hours are shown in Supplementary Figure 3. All three infected rabbit corneas showed thick mucopurulent discharge, severe conjunctivitis, dense neutrophil infiltration with liquefactive necrosis and hypopyon. SHG second harmonic imaging of the rabbit cornea infected *in vivo* revealed banded areas of PA localization that aligned with the orientation of the collagen lamellae (Fig. 6A–E). Alignment remained significant (R = 0.389 ± 0.171, P < 0.001) but the correlation between PA and collagen lamellar orientation was reduced since PA was only present in specific banded sections and not throughout the entire stroma. Interestingly, within the stroma, PA also aligned itself along what appeared to be a potential stromal nerve (Fig. 7).

**Figure 8:**
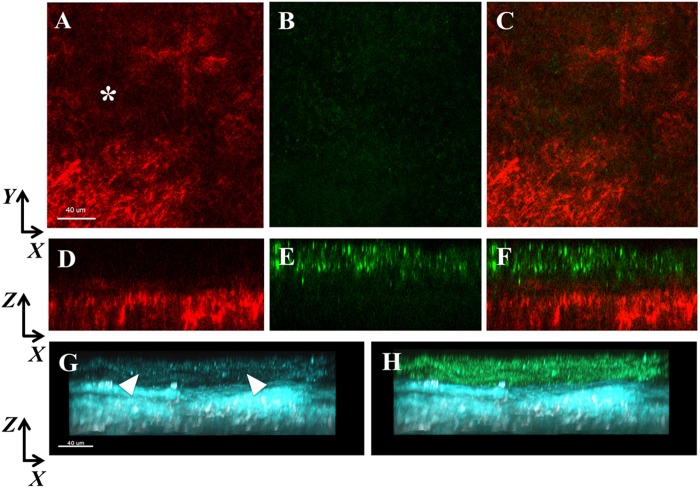
SHG forward signal in a dense infiltrate (corresponds to biomicroscopic image in Supplementary Figure 2C). (**A**) *XY* slice showing deterioration of the collagen SHG forward signal (red, asterisk). (**B**) No PA (green) was visible corresponding to the forward SHG signal. (**C**) Merged overlay. (**D**–**F**) *XZ* slices of images (**A**–**C**), respectively showing distinct regions of PA and the forward SHG signal. In areas of dense PA, the forward SHG signal was very weak to non-detectable. (**G**–**H**) Backward SHG signal (teal) showing loss of signal in apical region (arrowheads). In areas of dense PA, a weak backward SHG is visible. (**F**) Merged overlay. Scale bar: 40 μm.

SHG imaging in the densest region of the infiltrate revealed focal loss of the forward SHG signal indicating complete necrosis of the collagen (Fig. 8). The backward SHG signal in this region was largely degraded with only minimal signal detected.

## Discussion

This is the first study to demonstrate the co-alignment of PA and the collagen lamellae within the corneal stroma. The ability of PA to traverse along the collagen lamellae in the corneal stroma, as opposed to shear digestion of the collagen, suggests that the rapid spread of PA through the corneal stroma is aided in part by the unique arrangement of collagen. Unlike the sclera that has an irregular network of collagen or the limbus, which runs in tight circumferential bands, corneal collagen is oriented in a precise, linear fashion to facilitate the passage of light. This linear arrangement appears to facilitate the rapid spread of PA through this tissue and may explain why infectious keratitis typically occurs within the central and paracentral cornea. In contrast to this, the PA did not enter and traverse through the sclera, despite direct exposure at the scleral rim after excision.

Similar to the sclera, PA infection rarely occurs in the limbus. In concert with this, in our model none of the *ex vivo* or *in vivo* corneas demonstrated spread of the bacteria beyond the limbus. While these early data suggest that the non-ordered alignment of collagen in the sclera may provide a natural barrier to prevent infection of the sclera, other factors may contribute to the lack of spread through the limbus. This includes the tight-banded, ring-like circumferential collagen orientation in the limbal stroma, the size and density of the lamellar fibrils, differences in nutritional factors, and the presence of the limbal arcades. Further studies to expand upon the role of the limbus as a second line of defense against the spread of pathogens into the cornea are needed.

Importantly, the correlation between PA and collagen lamellae orientation noted *ex vivo* was also present in the *in vivo* rabbit contact lens model that lacked a penetrating wound through the basal lamina. The basal lamina acts as the final gatekeeper to corneal infection. Once through, PA continued on a linear path as it spread through the cornea. As the density of PA increased, the linear directionality was lost and eventually, as infection continued to worsen, subsequent liquefactive necrosis of the anterior cornea was associated with a loss of SHG signal generation. These data suggest that the use of SHG imaging may provide a new tool for monitoring disease progression and resolution during corneal infection. While a quantitative metric for monitoring disease resolution and residual scarring in the cornea is needed for the development of new therapeutics to both combat and mitigate disease, currently there is no commercial instrument that can be applied in human clinical practice. Thus, at this time, the use of this technique is limited to laboratory-based evaluation.

A previous group used SHG imaging to study infected human corneas *ex vivo*. In this study, the authors reported changes in the SHG signals indicating structural alterations in the cornea^36^. A limitation to this study was the absence of any fluorescence marker for the infecting bacteria, thus areas of bacterial spread could not be directly correlated to regions that lost SHG signals. A more recent study has investigated SHG changes in the rabbit cornea following infection with *Aspergillus fumigatus* and *Candida albicans*^35^. Interestingly, they also observed that some of the filamentous structures formed during infection appeared to align with collagen lamellae; however, directionality was not measured in this study. Finally, studies investigating PA in a 3D mouse model of infection also showed a regular linear arrangement of PA in the anterior stroma. However, in this study the authors used reflection microscopy to visualize the extracellular matrix as opposed to second harmonic generating imaging as used in this study. Without the corresponding use of SHG imaging to visualize the arrangement of collagen, no definitive conclusions regarding the relationship between PA and collagen were possible^38^.

Certain cell types have been shown to use contact guidance as a means to move within three dimensional tissue environments. In the wounded cornea, fibroblasts use the linear organization of the intrastromal collagen to facilitate migration through the tissue^37^. This form of contact guidance is thought to aid in repopulation of cells to the wounded area. In contrast to wound healing, dense collagen networks have been shown to inhibit contact guidance by immune cells, thus promoting tumor environments^40^. While this is the first study that reports the precise alignment of bacteria with fibrillar collagen as a mechanism to facilitate infection, further studies are needed to determine whether PA uses contact guidance to mediate PA spread through the corneal stroma or follows the path of least resistance by moving along the grooves in the collagen bundles. Additionally, experiments to investigate whether cytotoxic strains of *P. aeruginosa* and invasive mutants also display this unique method of traversal through the stroma are also required.

In summary, SHG imaging combined with multiphoton fluorescence microscopy provides a powerful tool for assessing microbial infections in the cornea. The strength of using SHG imaging combined with multiphoton fluorescence microscopy is illustrated here by the direct visualization of cells and architecture of the extracellular matrix (ECM). Using this technique, the data presented here suggests that PA uses the precise arrangement of collagen lamellae to facilitate rapid spread through the corneal stroma. Further studies using both cytotoxic and invasive bacterial strains are needed to confirm these findings.

## Methods

### Bacteria

*P. aeruginosa* strain 6487, an invasive clinical isolate that is stably conjugated to a green fluorescent protein (GFP)-expressing plasmid (pSMC2) containing a carbenicillin-resistant cassette, was used in this study (a gift of Dr. Suzanne Fleiszig, UC Berkeley). Bacterial stocks were maintained in tryptic soy broth containing glycerol at −80 °C. PA was cultured overnight on tryptic soy agar (Sigma, St. Louis, MO) containing 300 μg/ml carbenicillin (Sigma, St. Louis, MO) at 37 °C. A single colony was selected and sub-cultured on a tryptic soy agar slant containing 300 μg/ml carbenicillin overnight (approximately 18 hour) at 37 °C. PA was re-suspended in antibiotic-free RPMI (Rosswell Park Memorial Institute medium, Sigma, St. Louis, MO) and adjusted to an optical density of 0.300 (corresponding to a concentration of approximately 10^8^ CFU/mL) using a spectrophotometer (SmartSpec Plus, BioRad, Hercules, California). The same strain of *P. aeruginosa* was used for both *ex vivo* and *in vivo* experiments.

### Rabbit corneas *ex vivo*

A total of 60 whole rabbit globes were used in this study. Rabbit globes were purchased from Pel Freeze Biologicals (Rogers, AR). Three 75 μm deep incisional wounds were made using a diamond knife and corneas were then excised. The crystalline lenses were left intact to maintain proper corneal shape during culture with the apical side of the epithelium facing upwards. Corneas were inoculated with ~10^8^ CFU/mL in RPMI media and allowed to incubate for 2, 6, 18, 24 and 48 hours at 37 °C. Three corneas were used at each time point. Corneas in RPMI alone were used as controls at each time point (5 corneas, 1 per time point). At the indicated time points, corneas were removed and fixed in RNase-free 4% paraformaldehyde (Electron Microscopy Sciences, Ft. Washington, PA). Corneas were then cut into strips and mounted apical side down on coverslip bottom tissue culture dishes (MatTek Corporation, Ashland, MA) in a 50:50 v/v mixture of glycerol and phosphate-buffered saline (PBS). The experiment was repeated for a total of three independent times.

### Rabbit corneas *in vivo*

All animals were treated according to the ARVO statement for the use of animals in Ophthalmic Research. All procedures were approved by the Institutional Animal Care and Use Committee at the University of Texas Southwestern Medical Center, Dallas, Texas. A total of three female New Zealand white rabbits weighing 2.5–3.5 kg (Charles River Laboratories, Wilmington, MA) were used in this study. All rabbits underwent a partial nictitating membranectomy under anaesthesia to facilitate contact lens wear^15^. Using our previously reported contact lens rabbit model of infection, high oxygen transmissible rigid gas permeable contact lenses (tisilfocon A, Dk 160, Menicon, Nagoya, Japan) with an overall diameter of 14.0 and base curves ranging from 7.60–8.20 mm were inoculated with approximately 10^8^ CFU and allowed to incubate in a humidified chamber overnight at 37 °C. Rabbits were anesthetized with 1.5 mg/kg Xylazine (Anased, Shenandoah, IA) and 30 mg/kg Ketamine HCL (Ketaset, Ft. Dodge Animal Health, Ft. Dodge, IA) for the contact lens fitting. Lenses were placed on rabbit corneas and worn for 24 hours. Only one eye for each rabbit was fit with an inoculated contact lens (n = 3). The other eye served as a non-lens wearing control (n = 3). Rabbits wore the lenses until the development of a corneal ulcer.

After 24 hours of lens wear, all rabbits showed signs of corneal ulceration. Rabbits were anesthetized at this time point with 5 mg/kg Xylazine and 50 mg/kg Ketamine HCL. Lenses were removed using sterile tweezers and corneas were photographed using a Haag-Streit Slit Lamp (Haag-Streit Diagnostics, Mason, OH) equipped with a SONY digital camera (DSC-W90; Sony Corp., New York, NY, USA). Animals were euthanized by an intravenous injection of 120 mg/kg pentobarbital sodium (Euthasol, Virbac Animal Health, Ft. Worth, TX) followed by a bilateral thoracotomy. Corneas were removed, immediately fixed in 4% paraformaldehyde and mounted as described above.

### Second harmonic generation and multiphoton fluorescence imaging

Second harmonic generation and multiphoton fluorescence imaging was performed using a Leica SP8 confocal microscope (Leica, Heidelberg, Germany) equipped with a ultrafast Ti: Sapphire multiphoton laser (Coherent Chameleon Vision II, Coherent, Santa Clara, CA). An 880 nm wavelength was used for image generation. Forward and backward SHG signals and fluorescence were simultaneously acquired using a 25x water immersion objective lens with a 0.95 NA at a zoom of 2.00. Image sizes were 1024 × 1024 pixels. 50–100 μm thick image stacks were acquired with a z-step size of 1.0 μm.

### Image Analysis and Statistics

Image stacks were reconstructed three-dimensionally in Imaris (Bitplane, Concord, MA). 3D movies were made using the Leica Application Suites built-in software (Leica, Heidelberg, Germany). Orientation of PA with respect to collagen lamellae was analysed using the Directionality plug-in in ImageJ (National Institutes of Health, Bethesda, MD)^37^. For *ex vivo* corneas, directionality assessment was performed on the full 1024X1024 image. For *in vivo* models, smaller 256 × 256 regions were used to isolate the specific banded regions of PA infection. Statistical analysis was performed in SigmaPlot (Version 12.5, Systat Software, San Jose, CA). Linear regression was used to determine the correlation between PA orientation and collagen. The correlation coefficient from three independent *XY* slices in each stack were calculated and are expressed as mean ± standard deviation. To compare differences between two groups (in the 6 hour condition), a t-test was used. For comparisons between three groups (in the 18 and 24 hour conditions), a One-way ANOVA with appropriate post-hoc comparison test was used. A One-way ANOVA was also used to compare differences in the overall correlation coefficients between time points. P < 0.05 was considered statistically significant. All experiments were repeated a minimum of two additional times.

## Additional Information

**How to cite this article**: Robertson, D. M. *et al*. Second harmonic generation imaging of corneal stroma after infection by *Pseudomonas aeruginosa. Sci. Rep.* **7**, 46116; doi: 10.1038/srep46116 (2017).

**Publisher's note:** Springer Nature remains neutral with regard to jurisdictional claims in published maps and institutional affiliations.

## Supplementary Material

Supplementary Dataset

Supplementary Video 1

Supplementary Video 2
