# Supplementary material for: Second harmonic generation imaging of corneal stroma after infection by Pseudomonas aeruginosa
Source: Sci Rep. 2017 Apr 11;7:46116. doi: 10.1038/srep46116 (PMC5387403; doi:10.1038/srep46116)
Supplement: Supplementary Dataset [file srep46116-s1.doc]

Second harmonic generation imaging of corneal stroma after infection by *Pseudomonas* *aeruginosa*

Danielle M. Robertson, Nathan A. Rogers, W. Matthew Petroll and Meifang Zhu

From the Department of Ophthalmology

The University of Texas Southwestern Medical Center

**Supplementary Figures**

Supplementary Figure 1: SHG imaging of normal rabbit cornea. (A-C, top panels) *XZ* slices of the full thickness rabbit cornea. (A) forward signal (red); (B) backward signal (teal); and (C) merged imaged. (A-C, bottom panels) *XY* slices showing *en face* appearance of SHG signal in anterior cornea at the level of the dotted line in top panel. Scale bar: 50 μm.


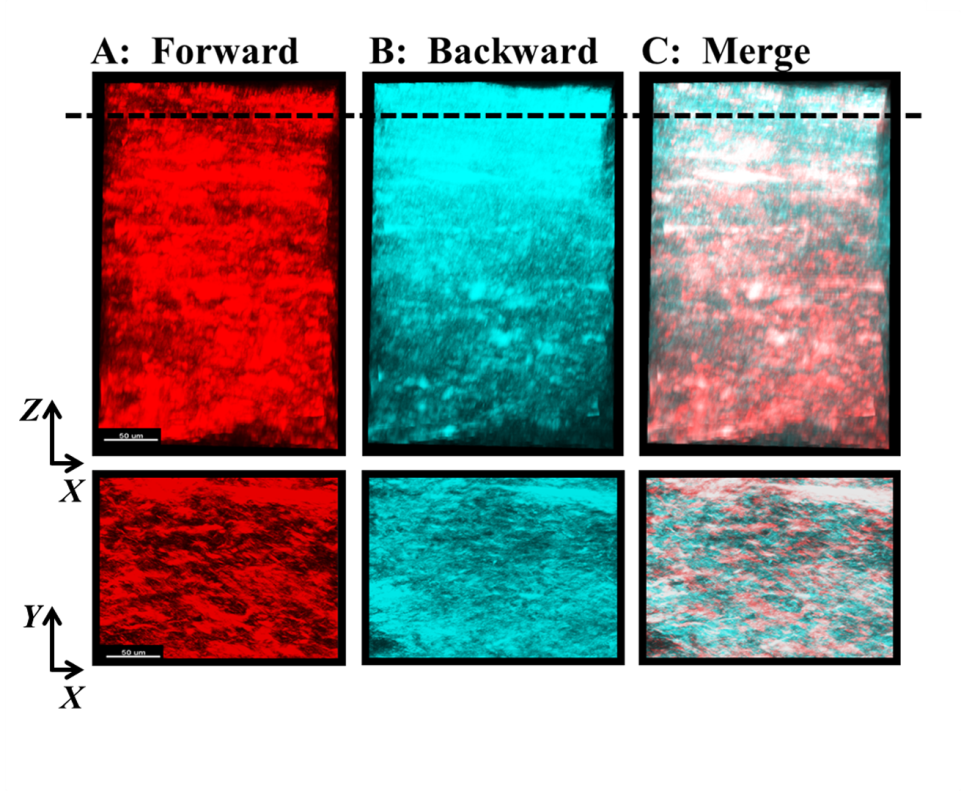


Supplementary Figure 2: Comparison between the correlation coefficient at different corneal depths and time points post-inoculation. (A) At 6 hours post-inoculation, the correlation coefficient was strongest anteriorly (plane B from Figure 3) and significantly reduced as you moved deeper into the stroma (plane C from Figure 3). This change was likely due to low levels of bacteria in the mid-anterior stromal region (P=0.015, n=3 images per region from a cornea, t-test). (B-C) There was no significant difference in the correlation coefficients at different depths in the stroma at either 18 or 24 hours. (D) Comparison of correlation coefficients demonstrated that the highest correlation between PA and collagen orientation was at the longer time points (18 and 24 hours). At 6 hours, the correlation coefficient was significantly reduced compared to 18 (p<0.001) and 24 (P<0.001) hours (n=6 images per cornea at 6 hours, n=9 images per cornea at 18 and 24 hours, One-way ANOVA).


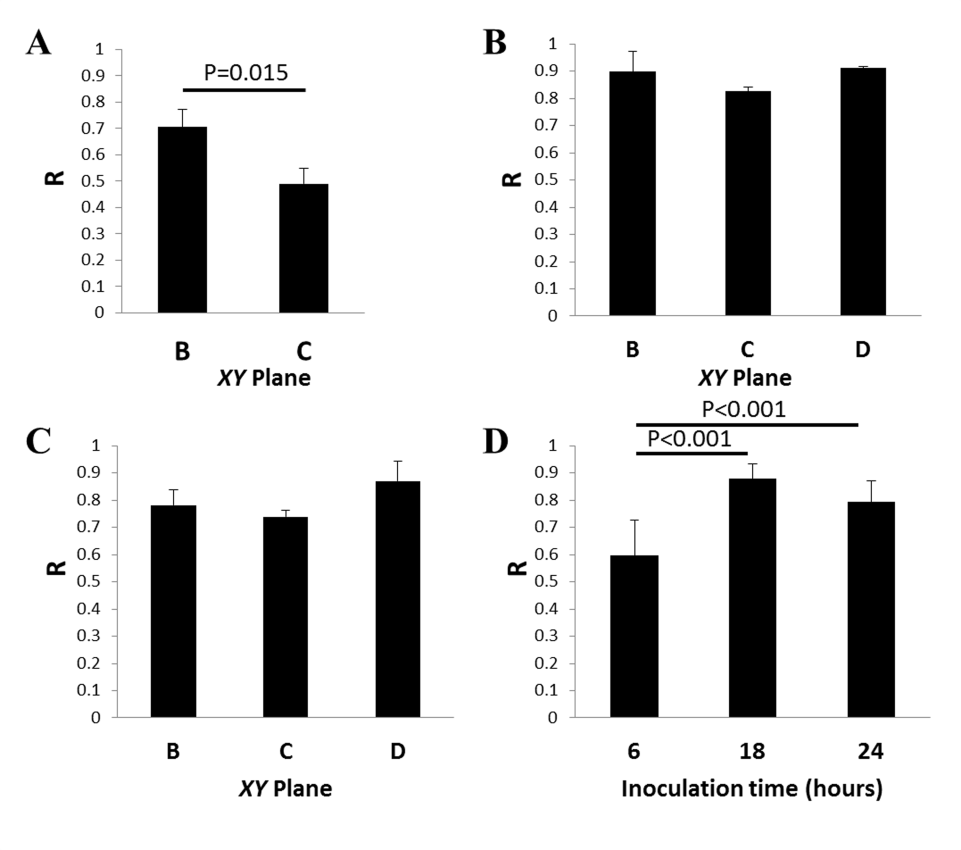


Supplementary Figure 3: Biomicroscopic images of the rabbit cornea *in vivo* after 24 hours of contact lens wear. (A) Normal rabbit cornea; (B&C) PA-infected rabbit corneas corresponding to Figures 7 (cornea B) and 8 (cornea C).


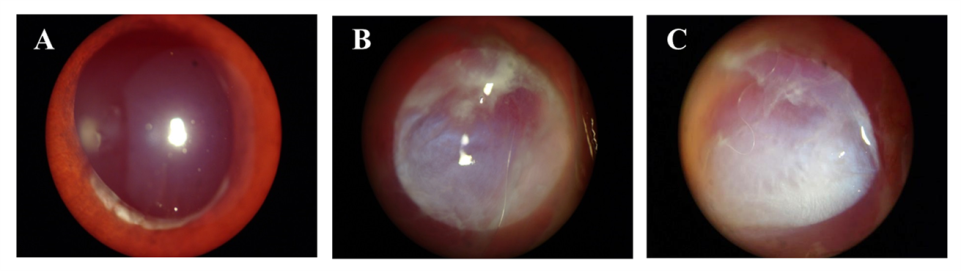


Supplementary Figure 4: 3D videos showing the relationship between the orientation of PA and the forward SHG signal. PA shown in green; forward SHG signal in red. (A) Higher PA-dense region; (B) lower PA-dense region.
